# Autism Spectrum Disorder Symptom Profile Across the RASopathies

**DOI:** 10.3389/fpsyt.2020.585700

**Published:** 2021-01-15

**Authors:** Marie-Maude Geoffray, Bruno Falissard, Jonathan Green, Browyn Kerr, D. Gareth Evans, Susan Huson, Emma Burkitt-Wright, Shruti Garg

**Affiliations:** ^1^Centre Hospitalier Le Vinatier, Bron, France; ^2^Division of Neuroscience and Experimental Psychology, Faculty of Biological Medical & Health Sciences, University of Manchester, Manchester, United Kingdom; ^3^CESP, INSERM U1018, Université Paris-Saclay, Villejuif, France; ^4^Manchester Academic Health Sciences Centre, Manchester, United Kingdom; ^5^Department of Child and Adolescent Mental Health, Manchester University NHS Foundation Trust, Manchester, United Kingdom; ^6^Manchester Centre for Genomic Medicine, Manchester University NHS Foundation Trust, Manchester, United Kingdom; ^7^Division of Evolution and Genomic Science, Department of Genomic Medicine, St Mary's Hospital, University of Manchester, Manchester, United Kingdom

**Keywords:** ASD, RASopathies, Noonan syndrome, cardio-facio-cutaneous syndrome, neurofibromatosis type 1, early intervention

## Abstract

Dysregulation of the Ras MAPK signaling pathway is implicated in the pathogenesis of autism spectrum disorder (ASD). The RASopathies, a group of disorders caused by mutations of the Ras/MAPK pathway genes, share many overlapping clinical features. Studies suggest a high prevalence of ASD in the RASopathies, but detailed characterization of the ASD profile is lacking. The aim of this study was to compare the ASD symptom profile of three distinct RASopathies associated with both gain-of-function and loss-of-function mutations: neurofibromatosis type 1 (NF1), Noonan syndrome (NS), and cardiofaciocutaneous syndrome (CFC). Participants were drawn from existing databases if they had a diagnosis of a RASopathy, met the criteria for ASD, and were able to communicate verbally. We compared the phenotypic profile of NF1 + ASD (*n* = 48), NS + ASD (*n* = 11), and CFC + ASD (*n* = 7) on the Autism Diagnostic Inventory (ADI-R) and the Autism Diagnostic Observation Schedule (ADOS). We found subtle but non-significant group differences with higher levels of social impairments and lower restricted repetitive behaviors in the NF1 group as compared with the NS and CFC groups. We observed group differences in developmental milestones with most severe delays in CFC, followed by NS and NF1. Our results suggest that despite developmental differences, the ASD profile remains relatively consistent across the three RASopathies. Though our results need confirmation in larger samples, they suggest the possibility that treatment and mechanistic insights developed in the context of one RASopathy may be generalizable to others and possibly to non-syndromic ASD associated with dysregulation of Ras/MAPK pathway genes.

## Introduction

Autism spectrum disorder (ASD) is a common neurodevelopmental disorder characterized by deficits in social communication, restricted repetitive behaviors, and sensory sensitivities. The phenotypic presentation and the genetic architecture of ASD is heterogeneous and complex which makes the discovery of pharmacotherapies inherently difficult (1). Nevertheless, important insights about underlying neurobiology of ASD has been gained through the study of single-gene disorders like Fragile X (FXS), tuberous sclerosis (TS), and neurofibromatosis 1 (NF1) which are associated with a high penetrance of ASD. Although hundreds of genes are implicated in ASD, studies using systems biology approaches suggest that these genes converge on a parsimonious set of downstream molecular pathways that are important for human brain development (2). Many of these downstream pathways are important for regulating protein synthesis, synaptic plasticity, and chromatic remodeling (3–5). More recent studies have implicated an important mechanistic link between ASD and the mitogen activated protein kinase (MAPK) signaling pathway which has a key role in regulating synaptic plasticity and memory (4, 6). The MAPK pathway is well known for its role in oncogenesis and is critically involved in the regulation of the cell cycle (7), but its role in neurodevelopment is also becoming clear (8–10).

The MAPK pathway is central in the pathogenesis of a group of disorders collectively known as the RASopathies that affect 1 in 1,000 live births (7). These disorders include neurofibromatosis type 1 (birth incidence 1 in 2–2,700) (11, 12), Noonan syndrome (NS) (birth incidence 1–2,500) (13), and its variants (including Noonan-like syndrome with multiple lentigines and Noonan-like syndrome with loose anagen hair), cardiofaciocutaneous syndrome (CFC), Costello syndrome (CS), and Legius syndrome. The RASopathies are multisystem disorders characterized by clinical features including cardiac malformations, facial dysmorphology, growth retardation, increased risk of cancer, and a wide spectrum of cognitive deficits (14). Clinical studies have established a substantial increased risk of ASD in NF1 (15), NS, and CFC (16–18) with estimates of about 30% in NF1 and Noonan syndrome and up to 90% in CFC including a male bias as seen in idiopathic autism. There is also a substantial prevalence of attention deficit hyperactivity disorder (ADHD) with estimates of up to 50% in NF1 and NS and even higher rates in CFC.

The MAPK pathway has an important role during neurodevelopment for critical regulation of cell cycle and maintenance of neural progenitor cells population that give rise to mature neurons that form synapses and neuronal circuits for normal cognition. Disrupted MAPK signaling during cortical development leads to abnormal progenitor proliferation and excitability thus affecting brain development and function (19). Pharmacological inhibition of MAPK pathway in animal models results in social deficits, impaired long-term potentiation, and impaired memory. NF1 animal models have altered ultrasonic vocalizations and deficits in social memory (20, 21). Mutations of genes both upstream and downstream of Ras result in activated MAPK signaling although via distinct cellular mechanisms (10). Loss-of-function mutations in NF1 result in hyperactive MAPK pathway which downstream preferentially affects GABAergic signaling resulting in the cognitive/social deficits. In NS, mutations of different genes result in gain of function which cause hyperactivation of MAPK pathway and increased excitatory neurotransmission downstream. CFC is caused by both loss-of-function and gain-of-function mutations of the BRAF gene and results in hyperactivation of the MAPK pathway and increased GABAergic signaling downstream. Despite these cellular differences, studies in animal models suggest similarities in electrophysiological and behavioral phenotypes. Indeed, treatment with HMG-CoA reductase inhibitors such as Lovastatin has been shown to reverse the cognitive phenotype in animal models of both NS and NF1 (22).

There has been much interest in the possibility of syndromic ASD causing patterns of autistic symptomatology based on the assumption that perturbations in certain biological pathways would lead to relatively constrained and more specific phenotypic outcomes (23, 24). Certain disorders such as Rett syndrome, Fragile X, or Williams syndrome are known for their characteristic autistic profile, for instance, symmetric hand stereotypies in Rett syndrome or poor eye contact in Fragile X (25, 26). Within the context of RASopathies, detailed characterization of the ASD phenotypic profile has been investigated in NF1 as compared with non-syndromic ASD, which showed lack of any characteristic “syndromic” specificity. ASD in NF1 is similar to non-syndromic ASD but with improved eye contact and fewer restrictive repetitive behaviors in NF1 (15). However, there have been no studies to date comparing the characteristic of ASD profile between different RASopathies. Given that both gain-of-function and loss-of-function mutations activate MAPK pathway, the aim of this study was to compare the developmental and autism symptom profiles in children with three RASopathies (NF1, NS, and CFC) and ASD. Based on the findings from animal models, our hypothesis is that despite the cellular differences, the ASD profile will demonstrate similarities across these disorders.

## Methods

### Participants

Participant data collected on children with NF1, NS, and CFC aged 4–18 years were available from three previously published independent studies (15, 16, 27). Two studies characterized the ASD prevalence in NF1, NS, and CFC. A further sample was drawn from a randomized controlled trial (RCT) of simvastatin in children with NF1. Participants included as part of the simvastatin RCT were recruited from regional genetic centers and through NF charity advertisements. All participants had detailed ASD assessments. Participants who met the following inclusion criteria were included in this study: (1) meeting ASD cut-off on the Autism Diagnostic Interview Revised (ADI-R); (2) scoring above the ASD cut-off on the Autism Diagnostic Observation Schedule-2 (ADOS-2); (3) having a minimal language by sentence, as confirmed by the use of a module 2, 3, or 4 ADOS-2. Children with “no or few words,” assessed with module 1 ADOS-2, were excluded from this study.

Out of 212 children with RASopathies in the datasets, 69 children with RASopathies and ASD met the inclusion criteria. Among these children with RASopathies and ASD, three children were non-verbal and excluded from the overall analyses as their profile are not directly comparable with verbal ASD. Thus, our final population consisted of 66 children with RASopathies and ASD.

### Measures

Behavioral phenotyping was done using the following standardized well-validated measures:

(1) The Wechsler Abbreviated Scale of Intelligence-II (WASI-II) was used to measure verbal intellectual functioning (VIQ), based on two subscales called Similarities and Vocabulary.

(2) The Autism Diagnostic Observation Schedule–second edition (ADOS-2) was used to measure current autism clinical profile. ADOS is a semi-structured, standardized observational assessment in communication, play, imaginative skills, and repetitive behaviors. It consists of four modules appropriate to different developmental age and expressive language skills. According to the ADOS manual, module 2 is for children of any age who use phrase speech but are not verbally fluent, module 3 is for verbally fluent children and young adolescents, and module 4 is for verbally fluent older adolescents. For the purposes of this study, we use all the items common to modules 2, 3, and 4. Scores on items range from 0 (no evident abnormality) to 3 (marked abnormality). We also used the subscale Social Affect domain (ADOS-SA) and Restrictive and Repetitive Behavior domain (ADOS-RRB), and the overall score (ADOS-composite) scored in Comparative Severity Score (CSS) (minimum–maximum = 0–10) (28). The CSS are calibrated scores derived from raw total scores. CSS of 1–3 corresponds to an ADOS classification of “Nonspectrum,” CSS of 4–5 “ASD,” and a CSS of 6–10 to “autism” [see Hus et al. (28) for more details].

(3) Autism Diagnostic Interview-Revised (ADI-R) (29) was used for detailed parent interviews. It measures the clinical profile throughout the development based on parent-reported observation gathered during a structured interview. The aim of the interview is to collect data about the most abnormal behavior exhibited in the past, either when the child was between 4 and 5 years old or “Ever” (“4–5 most abnormal/ever”). The ADI-R also allows for collecting data on early milestones and early general skills development (e.g., age of first phrases). It includes 87 items scored in range from 0 (no evident abnormality) to 3 (marked abnormality) except for the early general skills development which are scored in range of age of acquisition. First Walked unaided was considered delay after 18 months old and very delay after 36 months old. Daytime Bladder control and bowel control were considered delay for an age equal or superior to 36 months, and very delay after 60 months. Night-time Bladder control was considered delay after 48 months and very delay after 84 months. First single words were considered delay after 24 months and very delay after 60 months old. First phrases were considered delay after 36 months old and very delay after 60 months old.

Individual items (scored 0–3) were grouped together in 12 ADI-R subscores in the proposed algorithm which in turn provided three domains scores, which are Qualitative Abnormalities in Reciprocal Social Interaction (Domain A), Qualitative Abnormalities in Communication (Domain B), and Restricted, Repetitive, and Stereotyped Patterns of Behavior (Domain C). Each of the domains A, B, and C is composed of four subscores, correlating to specific ASD symptoms.

(4) Conners Parent Rating Scale (3rd edition, short form) measured ADHD symptomatology. The two domains, inattention and hyperactivity, were used in the study and presented in T-score. A significant clinical score was defined as greater than or equal to 65.

### Statistical Analysis

Data were analyzed in SPSS version 25 (SPSS Inc., Chicago, USA). Mean (M) and SD (or ratio for categorical variable) of each characteristic of the population were described. As ADOS items, domain and overall scores CS, items of early general skills development, ADI-R algorithm subscores, and ADI-R algorithm domains mean scores were not normally distributed in each of the RASopathies, non-parametric analyses were used. Summary statistics including group means and CIs are presented.

Graphs described the current clinical profile with ADOS items, autistic clinical Profile by the “4–5 most abnormal/ever” subscores of ADI-R, and early general skills development. In an exploratory analysis, we compared the clinical profile, currently, the most abnormal at 4–5 years old or “ever” and throughout early development, between the three groups of children with RASopathies using an independent-samples Kruskal–Wallis test. A two-tailed *p*-value of < 0.001 was considered significant after Bonferroni correction for multiple comparisons.

## Results

### General Characteristics of the Population (Table 1)

This sample included 48 children with NF1 + ASD, 11 children with NS + ASD, and 7 children with CFC + ASD. The mean age of the sample was 8.9 (*SD* = 2.7) years with no significant difference in age across the three groups (*p* = 0.079). There were more males than females in all the three groups: the ratio of males to females was 3:1 in NF1, 4.5:1 in NS, and 1.3:1 in CFC. The mean verbal IQ across the current population of RASopathies + ASD was 87.0 (*SD* = 13.8). The CFC group had the lowest VIQ, but there were no significant differences across the groups (*p* = 0.853). Only 27.2% of children with NF1 had a special educational statement at school as compared with 45% in the NS and 85% in the CFC groups.

**Table 1:** Characteristics of the children with neurofibromatosis type 1 (NF1) + ASD, Noonan syndrome (NS) + ASD, and cardiofaciocutaneous syndrome (CFC) + ASD.

	**NF1 + ASD**	**NS + ASD**	**CFC + ASD**	**Kruskal–Wallis test *p***
*N*	48	11	7	
Mean age (95% CI)	8.4 (8.3–8.5)	9.3(8.8–9.8)	11.2 (10.5–11.9)	0.079
Gender (M/F)	36/12	9/2	4/3	
Mean VIQ (95% CI)	88.7 (88.1–89.3)	89.1(87.5–90.7)	79.8 (73.6–85.9)	0.853
Statement of special educational needs	12/48	5/11	6/7	
**ADOS current autism behavior**				
Mean ADOS-SA CSS (95% CI)	7.3 (7.2–7.4)	6.4(6.1–6.7)	5.3 (4.5–5.9)	0.078
Mean ADOS-RRB CSS (95% CI)	5.0 (4.9–5.1)	6.5(6.2–6.8)	8.3 (8.0–8.5)	0.009
Mean ADOS-composite CSS (95% CI)	6.8 (6.7–6.9)	6.4(6.2–6.6)	6.4 (5.9–6.9)	0.652
**ADI “4–5 most abnormal/ever” autism behavior**				
Domain A: Mean Qualitative Abnormalities in Reciprocal Social Interaction (95% CI)	18.9 (18.7–19.1)	17.4(16.4–18.4)	18.4 (17.2–19.6)	0.743
Domain B: Mean Qualitative Abnormalities in Communication (95% CI)	13.7 (13.5–13.9)	14.8(14.2–15.4)	14.7 (14.1–15.2)	0.895
Domain C: Mean Restricted, Repetitive, and Stereotyped Patterns of Behavior (95% CI)	5.8 (5.7–5.9)	4.7(4.1–5.3)	5.6 (5.5–5.7)	0.649
**Conners scale for ADHD**				
Mean inattention (SD) (95% CI)	75.7 (75.2–76.2)	73.1(71.2–75.0)	80.7 (78.5–82.9)	0.460
Mean hyperactivity (SD) (95% CI)	74.1 (73.5–74.7)	71.8(69.2–74.3)	74.3 (71.4–77.2)	0.923

*Mean and CIs of the analysis with Kruskal–Wallis test are presented (p < 0.001)*.

NF1 was inherited in 24 out of 48 cases. In the Noonan syndrome group, six had a *PTPN11* mutation, one in RIT 1, and one SHOC-2 mutation; mutation status was unknown in three children. In CFC, six had a BRAF mutation and mutation status was unknown in one child.

On the ADI-R, the NF1 and CFC groups had higher levels of reciprocal social interaction impairments and restricted repetitive behaviors as compared with the NS group, but there were no significant differences between the groups. On the ADI-R communication sub-scale, NS and CFC groups had higher communication impairments as compared with the NF1 group but group differences were not significant. Similarly, on the ADOS, there were no significant differences in overall clinical severity scores. Sub-scale analyses showed that NF1 group had higher impairments in social affect as compared with NS and CFC group but lower levels of restricted repetitive behaviors. The CFC group showed overall higher RRBs, but group differences were not significant (*p* = 0.009). On the parent-reported Conners questionnaire, no mean differences were observed on the inattention (*p* = 0.460) or hyperactivity subscales (*p* = 0.923). However, ADHD symptoms were comorbid in 68.2% children with NF1 + ASD, 68.2% NS + ASD, and 85.7% with CFC + ASD.

### Detailed Characterization of the ASD Profile

#### Current Clinical Profile With ADOS Scores

**Figure 1:**
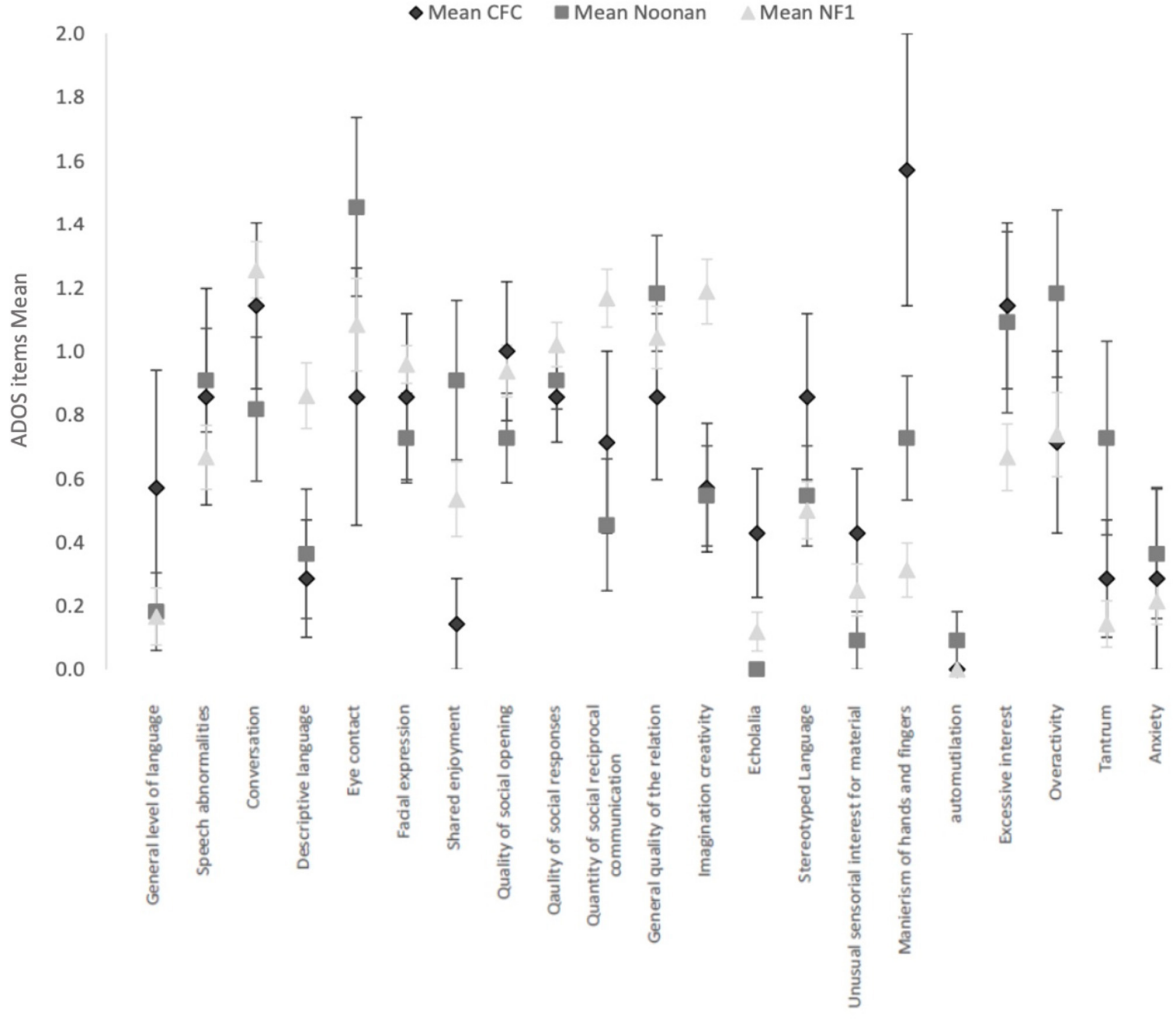
Current clinical profile with ADOS items (common across modules 2, 3, and 4) in the 3 RASopathies, CFC, Noonan Syndrome and NFl. Mean and Standard Error are represented for each item.

Figure 1 shows the mean and SEs on each of the ADOS items score common across the three ADOS modules. Comparative analysis of the ADOS items across the RASopathies using the independent-samples Kruskal–Wallis test showed no significant differences after accounting for multiple comparisons. The NF1 group had higher mean scores for difficulties in imagination and creativity (*p* = 0.004), in use of gestures (*p* = 0.01), and quantity of Social reciprocal communication (*p* = 0.005), but three RASopathy group differences were not significant.

The CFC group had higher mean scores for hand and finger mannerisms (CFC *M* = 1.6, SD = 1.1; NS *M* = 0.7, *SD* = 0.6; NF1 *M* = 0.3, *SD* = 0.6), but the group differences were not significant (*p* = 0.001). Similarly, the CFC group showed higher scores for echolalia, but the overall group differences were not significant (*p* = 0.02).

In the ADOS items measuring general behavior as observed directly by the professional during the ADOS, we observed a more frequent occurrence of tantrum symptoms in NS (*p* = 0.02).

#### Clinical Profile by the “4–5 Most Abnormal/Ever” ADI-R Scores

**Figure 2:**
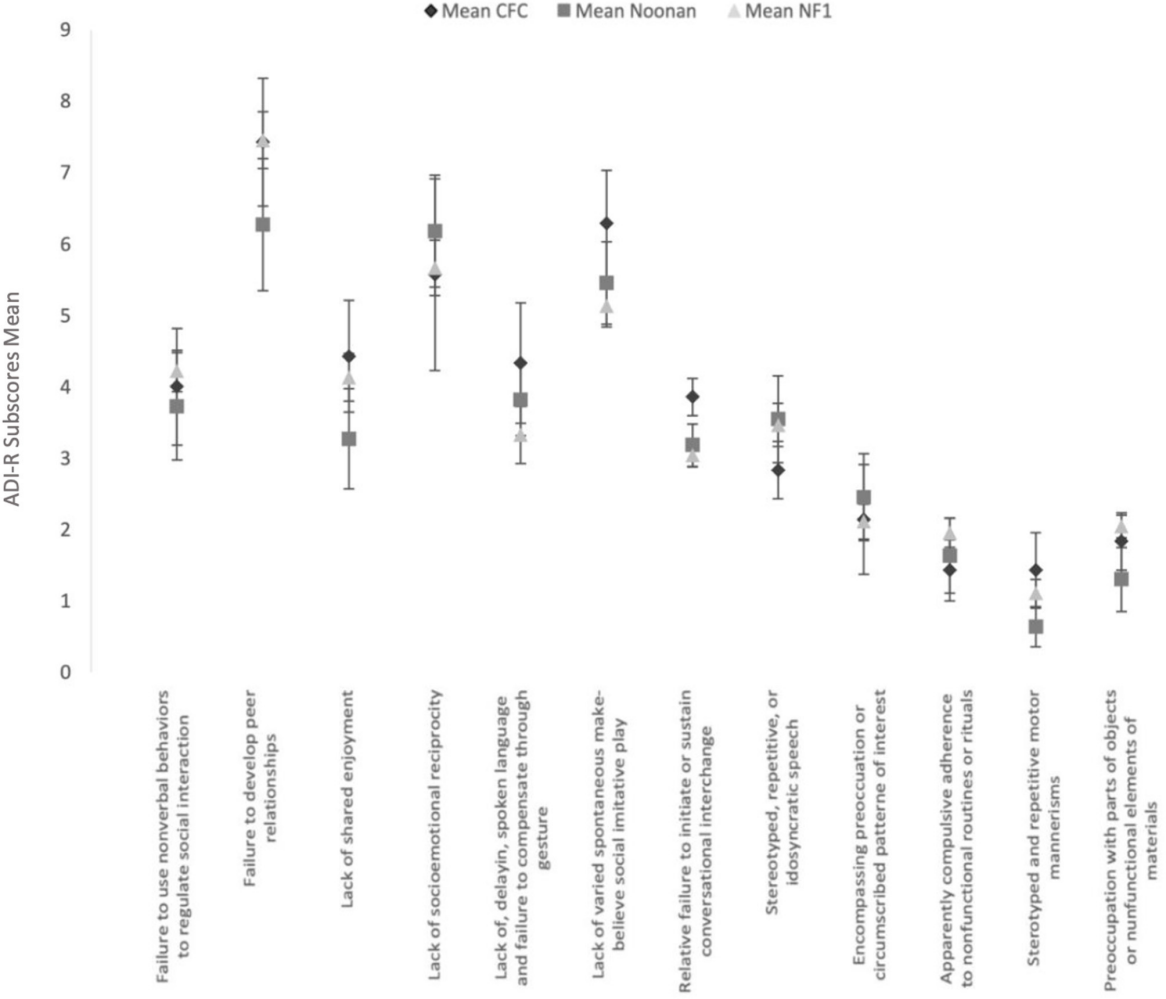
Clinical profile by the “4–5 most abnormal/ever” AD I-R subscores ADI-R subscores in the 3 RASopathies. Mean and Standard Error are presented for each subscores.

Figure 2 shows the mean and SEs of the algorithm subscores of ADI-R, based on the parent report of symptoms at the 4–5-year time window or “ever.” Comparative analysis of the ADI-R subscores shows striking similarities across the three groups and overall similarity to the phenotypic profile on the ADOS. Using the independent-samples Kruskal–Wallis test, there were no significant differences observed between the three groups.

#### Early Concerns and Milestone Development

**Figure 3:**
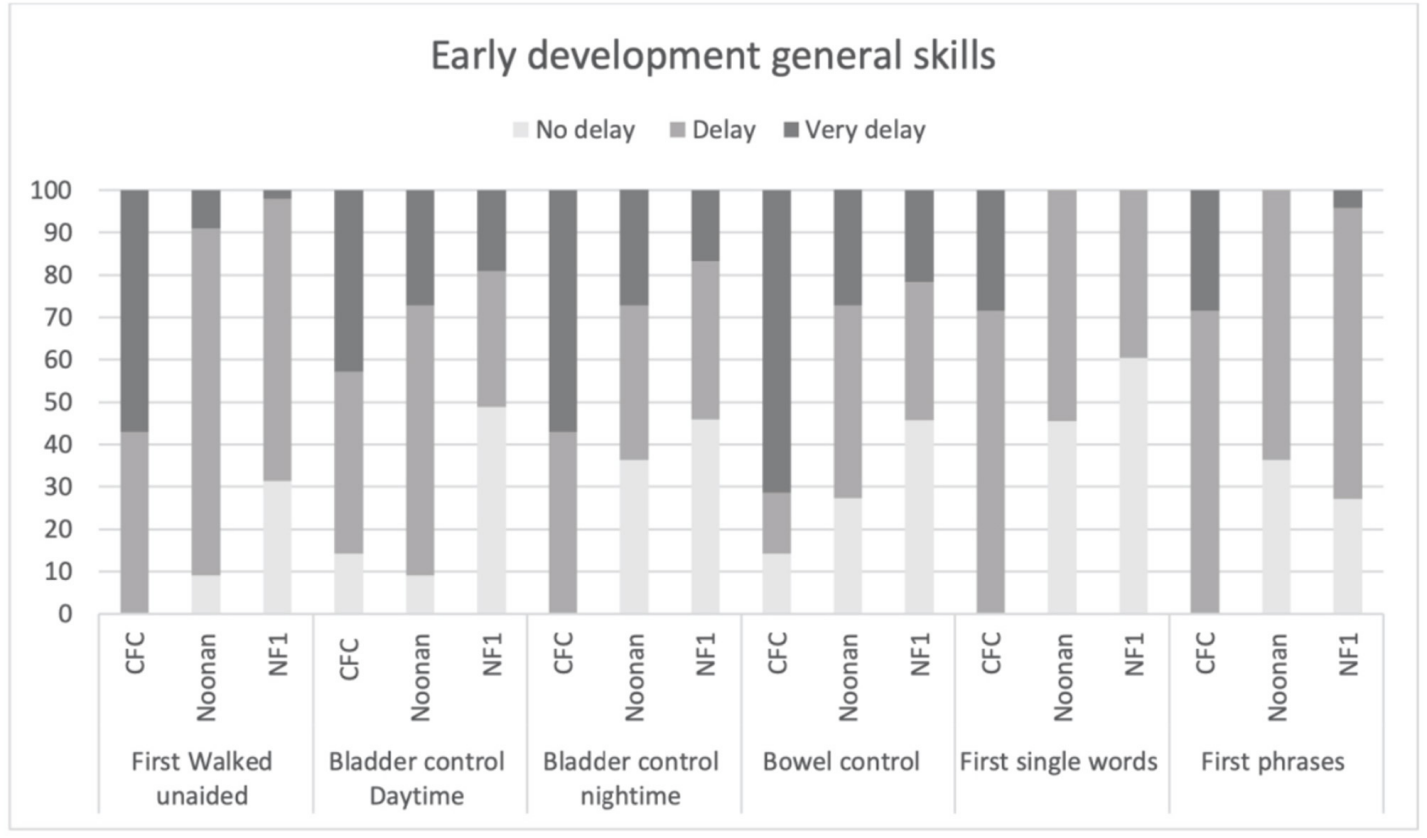
Age of acquisition early developmental milestones based on the parent structured interview of ADI in CFC, NS, and NFl.

Figure 3 shows the clinical profile of early general skills development. Overall, as would be expected, the CFC group showed more delayed development of motor and language milestones. Specifically, 100% of the CFC group, 90.9% of NS, and 64.6% of the NF1 group showed delays in walking unaided (defined as unable to walk unaided after 18 months) (*p* = 0.001). Similarly, 85.8% of the CFC group and 90.9% of the NS group showed delays in daytime bladder control (defined as no daytime continence after 36 months) as opposed to 51% in the NF1 group, but overall group differences were not significant (*p* = 0.042). Night-time bladder control was delayed (>48 months) in 100% CFC, 63.7% NS, and 54.2% in NF1 (*p* = 0.023). Acquisition of bowel control was delayed (>36 months) in 85.7% in CFC, in 72.8% in NS, and in 54.3% in NF1 (*p* = 0.051). First single words were delayed (>24 months) in 100% in CFC, 54.5% in NS, and in 39.6% in NF1 (*p* = 0.002). First phrases were delayed (>36 months) in 100% of participants with CFC, 63.6% with NS, and 73% with NF1 (*p* = 0.041).

Moreover, 100% of the parents of children with CFC reported that they first noticed that something was not quite right in language, milestones, or behavior before 3 years of age (ADI-R item 2) as compared with 36.4% in NS and 27.1% in NF1. Interestingly, only 14.3% of parents of children with CFC, 27.3% with NS, and 16.7% with NF1 felt the quality of their child's behavior was indicative of autism before 3 years old. Regression of skills, which is a common feature in autism, was not reported in CFC but reported by 6.4% of parents in the NS group and 9.1% in the NF1 group.

## Discussion

To our knowledge, this is the first study to compare the developmental and core ASD symptom profiles in children with RASopathies and ASD. In this cross-syndrome study, our results suggest subtle but non-significant differences in ASD symptom profiles with higher reciprocal social communication impairments but lower levels of restricted repetitive behaviors in the NF1 group as compared with the NS and CFC groups. Further, we found substantial differences in overall level of developmental delay and early developmental trajectory. Previous studies have noted the increased incidence of ASD in the RASopathies (15, 18, 30), but detailed characterization of the ASD profile has been lacking or has been based on brief parent-reported measures. Our results confirm previously reported similarities between NS and NF1 in levels of social competence based on parent report measures (30).

Although we did not include a non-syndromic ASD sample in our study, the overall ASD comparative severity scores (CSS) in the RASopathies show similarities to non-syndromic ASD. Thus, in two large cohorts of non-syndromic ASD, the mean CSS on the ADOS social affect and RRB were comparable with the RASopathies in this study. In one clinic-based prospective cohort (*n* = 1,415, aged 2–16 years), the mean (SD) of ADOS-SA CSS was 7.21 (2.17) and ADOS-RBB CSS 7.39 (2.19) (28). In another large US-wide clinic referral cohort from the Simon Simplex Collection (*n* = 2,509, aged 6–16 years predominantly with a level module 3 ADOS), the mean (SD) of ADOS-SA CSS was 7.22 (1.89) and the mean ADOS-RBB 7.66 (2.01) (31). A notable exception to this pattern of similarity is in NF1, where slightly lower levels of RRBs are observed (3) as compared with non-syndromic ASD populations (28, 31). Further as in non-syndromic ASD, we find a male preponderance of ASD across the three RASopathies although the sex differences are reduced in CFC possibly due to the lower intellectual ability in that group. In genetic disorders such as tuberous sclerosis or Cornelia de Lange associated with intellectual impairments, the ASD male bias is not seen (32). Further, our data suggest that ASD in RASopathies is highly comorbid with ADHD (54.5–85.7%), higher than in non-syndromic ASD where ASD is comorbid with ADHD in approximately 30% of the population (33). Presence of comorbidities such as ADHD and anxiety significantly increases the autism symptoms and is associated with poorer adaptive skills (34).

We also report for the first time detailed early developmental profiles based on parent interview. Overall, the CFC + ASD group are more delayed in acquiring early developmental milestones than the NS + ASD or NF1 + ASD groups. A large natural history study of 151 individuals with NS reported delays in both motor and language milestones with average age of walking unsupported at 21 months and use of simple two-word phrases at 31 months (35). Similarly, language and motor milestones delays have been reported in NF1 (36). Although these results are based on parental interview and thus subject to recall bias, they indicate that despite the differences in developmental milestones, the ASD profile and severity scores across the groups remain consistent. This suggests that the ASD phenotype in RASopathies does not track simply with the level of cognitive impairment as suggested by other studies reported in the literature (37).

A significant limitation of this study is the unequal sample sizes across the three disorders and a small simple size for the NS (*n* = 11) and CFC (*n* = 7) which may result in a type 2 error, that is, significant differences may be underreported. These may limit the generalizability of these findings, but larger sample sizes may be difficult to recruit given that these are rare genetic disorders. A further limitation may be the sampling frame and recruitment differences between the three groups. The CFC and NS samples were recruited via clinics and self-referrals through charity advertisements (16). The NF1 sample was recruited as part of two different studies—a population-based study of ASD ascertainment and a clinical trial (15, 27). However, participants included in this study were chosen across the research databases based on meeting a set of defined ASD criteria thus minimizing any biases related to recruitment differences. We excluded three non-verbal children as the ADOS non-verbal assessment module is significantly different to the modules for verbal children. Lastly, we did not have the physical phenotypic data for the population which may impact the cognitive phenotype. The field needs larger samples to confirm our results, and future investigations should consider genotype/phenotype correlation in the context of broader ASD risks. Given the difficulty of examining a large sample for any individual investigator, we recommend multisite studies as well as data sharing and integrative data analyses (38).

The strengths of this study include the careful ASD phenotyping based on the detailed parent-rated interviews (ADI-R) for the past behavior and, based on a direct observation, researcher rate (ADOS) for the current behavior allowing a careful examination of the phenotype across the three RASopathies.

Why are the results of this study important? ASD is a behaviorally defined syndrome with a high variability in the phenotypes across the spectrum. An increasing number of rare genetic variants are being implicated (>2,000 genes implicated) and highlight the complexity of genetic architecture and heterogeneity of ASD (2). Embryonic development is characterized by careful regulation of key cellular processes controlled by a limited number of key signaling pathways including the Ras-MAPK pathway which is highly expressed during fetal brain development. These pathways operate during development at different time points and different regions eliciting specific cellular responses. MAPK pathway has been implicated in non-syndromic ASD and hypothesized to be a point of convergence of many ASD risk genes (4). Using leukocyte transcriptomic analyses, a recently published study of toddlers with ASD discovered that the degree of dysregulation in key signaling pathways included Ras-MAPK, PI3K-AKT, and WNT correlates with ASD severity symptoms (39). The results of this study, although preliminary, suggest that despite the differences in cellular mechanisms, the downstream ASD phenotype may be consistent across the three RASopathies. Our results suggest that mechanistic and treatment insights gained from studying one RASopathy may be applicable to other RASopathies. Although we did not include a non-syndromic ASD group, overall ASD severity scores in RASopathies also show similarities to non-syndromic autism. Although many ASD risk genes converge downstream on the Ras-MAPK pathway, future studies investigating the ASD symptom profile in the RASopathies to non-syndromic ASD will be important to understand whether treatment insights from the RASopathies may be generalizable to non-syndromic autism (40). Ultimately stratifying ASD populations by the underlying key biochemical pathway affected may provide an insight into mechanisms and strategy for treatment discovery.

From a clinical perspective, our results provide important clinical knowledge about the early developmental time period in the RASopathies and highlight that despite the differences in developmental delays across the RASopathies, the ASD phenotype is an important cause of morbidity across all the disorders.

## Data Availability Statement

The raw data supporting the conclusions of this article will be made available by the authors, without undue reservation.

## Ethics Statement

The studies involving human participants were reviewed and approved by the ethics committees Greater Manchester Research Ethics Committee (reference 10/H1003/77 and 11/NW/0838). Written informed consent to participate in this study was provided by the participants' legal guardian/next of kin.

## Author Contributions

M-MG and SG built the protocol for this study, carried out the results and wrote the paper. BF carried out the statistical design. JG, BK, SH, EB-W, and DE, proof read, participated in the writing, and gave feedback. All authors contributed to the article and approved the submitted version.

## Conflict of Interest

The authors declare that the research was conducted in the absence of any commercial or financial relationships that could be construed as a potential conflict of interest.
